# Niche-dependent forest and savanna fragmentation in Tropical South America during the Last Glacial Maximum

**DOI:** 10.1038/s44185-024-00056-4

**Published:** 2024-09-11

**Authors:** Douglas I. Kelley, Hiromitsu Sato, Michaela Ecker, Chantelle A. Burton, João M. G. Capurucho, John Bates

**Affiliations:** 1 UK Centre for Ecology & Hydrology, Wallingford, Oxfordshire, UK; 2 Department of Earth Sciences, University of Toronto, Toronto, ON Canada; 3 Ontario Forest Research Institute , Ontario Ministry of Natural Resources, Ontario Sault Ste. Marie, Canada; 4 Institute of Prehistoric and Protohistoric Archaeology, Kiel University, 24118 Kiel, Germany; 5 Met Office, Fitzroy Road, Exeter, UK; 6 Coordenação de Biodiversidade, Instituto Nacional de Pesquisas da Amazônia (INPA), Manaus, Av. André Araújo, 2936 AM Brazil; 7 Departamento de Zoologia, Universidade Federal de Juiz de Fora, Minas Gerais, Brazil; 8 Integrative Research Center, Field Museum of Natural History, Chicago, IL USA

**Keywords:** Biogeography, Climate and Earth system modelling, Palaeoecology, Climate-change ecology

## Abstract

The refugia hypothesis, often used to explain Amazonia’s high biodiversity, initially received ample support but has garnered increasing criticism over time. Palynological, phylogenetic, and vegetation model reconstruction studies have been invoked to support the opposing arguments of extensive fragmentation versus a stable Amazonian Forest during Pleistocene glacial maxima. Here, we test the past existence of forest fragments and savanna connectivity by bias-correcting vegetation distributions from a Dynamic Vegetation Model (DVM) driven by paleoclimate simulations for South America during the Last Glacial Maximum (LGM). We find evidence for fragmented forests akin to refugia with extensive tropical humid forests to the west and forest islands in central/southern Amazonia. Drier ecosystems of Northern Llanos, Caatinga and Cerrado may have merged into continuous savanna/grasslands that dominated the continent. However, our reconstructions suggest taller, dense woodland/tropical savanna vegetation and areas of similar bioclimate connected disparate forest fragments across Amazonia. This ecotonal biome may have acted as a corridor for generalist forest and savanna species, creating connectivity that allows for range expansion during glacial periods. Simultaneously, it could have served as a barrier for specialists, inducing diversification through the formation of ‘semi-refugia’.

## Introduction

Determining what mechanisms drive the underlying richness of Amazonian biodiversity is one of the longest and most prominent debates in ecology and evolutionary biology^1^. Haffer’s refugia hypothesis is perhaps the most influential^2^, describing a potential set of past events to generate the observed diversity of birds in the Amazonian rainforest. The refugia hypothesis posits that cooler, drier conditions associated with glacial periods fractured the continuous closed-canopy moist forest. The remaining pieces of forest, or “refugia”, were isolated from one another by tracts of savanna vegetation. According to the hypothesis, fragmentation impeded gene flow between inhabitant biota and populations, driving diversification over long timescales. Refugia would reconnect during interglacial periods, and the newly speciated biota would expand their ranges. Expansion of open vegetation would also allow for range expansion and gene flow between distant populations of savanna species through the formation of corridors^3,4^.

Though the refugia hypothesis has an enduring influence on the study of biodiversity evolution across Amazonia, palaeoecological studies^5^ and genetic data for many lineages have been interpreted as counterevidence in terms of the pattern of habitat change and timing^6^. This led to the development of alternative hypotheses to explain past vicariance leading to diversification, such as emphasising the role of rivers in isolating populations^7,8^.

Concerning landscapes, the question of stability remains central to research efforts in modern, future, and palaeoecological contexts. Early climate modelling efforts for Amazonia during the LGM showed a stable rainforest, with savannafication only occurring on its borders^9,10^. Other major studies supported by palynological data also concluded a similar result. However, these studies lacked fundamental interactions between processes such as CO_2_ deprivation (effects of low CO_2_ on vegetation), climate and fire - critical in transforming the forest into savanna^10^. Palynological evidence of a stable Amazonia is also inconclusive based on the limited number of suitable sampling sites and rarity of appropriate data, with the most suitable cores being in its margins^11^.

**Fig. 1:**
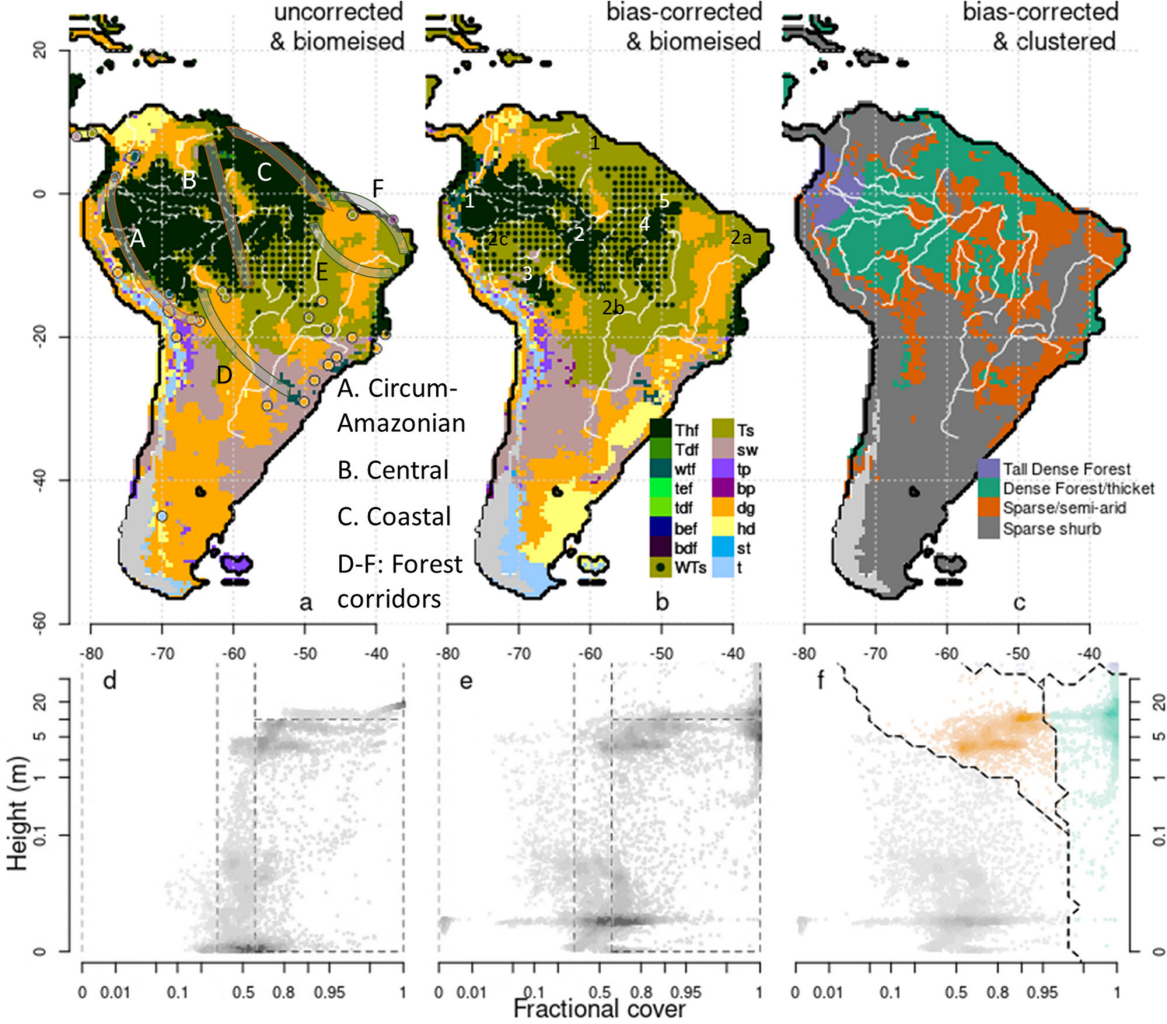
Biomes reconstruction during the LGM (left) as modelled in Sato et al^10^. (middle) using bias correction and (right) using clustering, driven by an ensemble of LGM climate reconstructions. Dots in **a** represent the pollen core locations used to correct reconstructions; colours in a and **b** indicate the biome reconstructed using pollen spectra. Biomes are: Thf, tropical humid forest; Tdf, tropical dry forest; wtf, warm temperate forest; tef, temperate evergreen forest; tdf, temperate deciduous forest; bef, boreal evergreen forest; bdf, boreal deciduous forest; Ts, tropical savannah; sw, sclerophyll woodland; tp, temperate parkland; bp, boreal parkland; dg, dry grass/shrubland; hd, hot desert; st, shrub tundra; t, tundra. WTs shows Woodland/tall savanna (not considered in Sato et al.) are shown with green dots on an olive background. Points in d-f correspond to individual grid cells in each map above. Colours in **d** and **e** indicate biomes, divided by total vegetation cover (x-axis) and vegetation height (y-axis) – dark grey is forests, green is savanna and parkland, red is grass and shrub, and light grey is arid. Colours in **c**, and **f** correspond to the clustering of **f**. A-C show potential savanna and D-F forest corridors proposed in the literature^12^. Small numbers 1-3 in **b** correspond to different (in white) forest and (in black) savanna fragments.

Sato et al. used a more comprehensive approach to reconcile the sparse and irregularly distributed palynological data with continuous theory-based model reconstructions of past vegetation^10^. They showed that modelled dynamic vegetation (DVM) simulations with the highest agreement to available pollen cores suggest widespread savannafication, contrary to past theories of a stable Amazonian Forest. Their analysis also revealed that in addition to drier glacial climate conditions, the combined effects of CO_2_ deprivation and the LGM fire regime may have driven these changes from forest to open, grassy biomes. These reconstructions also provided model evidence for savanna corridors. Three savanna corridors have been hypothesised to link currently disjointed savanna populations north and south of Amazonia^3,12^ (Fig. 1a). The circum-Amazonian corridor may have run along the western core of Amazonia along the Andes. The central corridor may have run through the more seasonal forest in the east. The coastal, or Atlantic corridor, has been hypothesised to have run from along the eastern coast. Sato et al.’s reconstructions feature central and circum-Amazonian savanna corridors and an extensive but partially fragmented forest (Fig. 1), but with forest that was still much more intact than suggested by Haffer’s refugia.

However, Sato et al.^10^ fell short of fully integrating model output and empirical data. While they used palynological data to interpret model reconstructions, assess model skill, and evaluate the likelihood of fire and CO_2_ deprivation contributing to vegetation distribution changes, they did not directly incorporate it into the reconstructions themselves. Even in simulations that agreed most closely with pollen data, there were still several points of disagreement (Fig. 1a). Namely, points the model predicted would have been forests were savannas according to pollen records. Statistical bias-correction - the process of improving model reconstructions to match observed data based on the disagreement between model and observation would improve the accuracy of the reconstructions.

In our study, we utilise bias-correction to directly incorporate empirical palynological data into dynamic vegetation models, enabling a better representation of past vegetation distributions and connectivity across Amazonia during the Last Glacial Maximum. We then examine the refugia hypothesis in light of these improved reconstructions, investigating the number of forest and savanna fragments across the regions. Additionally, we explore the differences in bioclimate and vegetation composition in the areas between forest and savanna fragments as a proxy for understanding how broad niches would have needed to be to maintain ecosystem connectivity. Finally, we use a clustering technique to identify areas of bioclimate stability and regions of rapid bioclimate transition, providing further insights into connectivity and fragmentation.

### Fusing proxy data and model output

In this study, we used reconstructions generated by Sato et al.^10^, who drove a fire-enabled DVM^13,14^ with four global climate model (GCM) outputs from the LGM^15^. The model outputs were growing degree days (GDD; summed daily mean temperature for temperatures above 5°C), fractional projected cover of vegetation (FPC), vegetation height, and evergreen, deciduous, tropical and temperate cover as a fraction of vegetation^10^ (Supplementary Figs. 2–7). A fifth model reconstruction was driven by the average of these bioclimates from the other four reconstructions and was found in Sato et al. to best match the collected pollen record^10^ (see methods). We, therefore, focus our results on this reconstruction.

We analyse the results in three ways:

1. Using a biomisation scheme adapted from Prentice et al.^16^ and Ciais et al.^17^ to translate raw model bioclimatic output to biome categorisation (Supplementary Fig. 1; see “biomisation ” in methods). The scheme defines each biome by bioclimatic thresholds, with forests and savannas having FPCs greater than 0.6. Savannas have a height of less than 10 m. To explore the differences in bioclimate and vegetation composition in the boundary between forest and savanna, we introduce a new woodland/tall savanna with FCP > 0.6 that is between 5 and 10 m in height. We divided Forest and Savanna biomes into the remaining biome categories using GDD, phenology and the tropical vs. temperate vegetation type ratio. We adapted some of the definitions in the scheme to allow us to quantify the distance between modelled and proxy measurements of biomes across all variables required for bias-correction in the next step.
2. We use a bias correction method to integrate empirical palynological data directly into our DVM output. We run this through the biomisation scheme to find the number of locations of simulated forest fragments that may have matched refugia. The bias-correction method shifted the vegetation cover, composition, height, and GDD DVM output to match the closest boundary (Supplementary Table 1) of the corresponding biome of each of 42 pollen-core observations (mapped in Fig. 1) taken from Marchant et al.^18^. These were then used as anchor points when we extrapolated this correction between pollen-core locations to produce continuous spatial reconstructions of past vegetation consistent with pollen records. See “bias-correction” in methods for more information.
3. We use a k-mean clustering technique similar to Sidoumou et al.^19^ on bias-corrected FPC and height maps to map areas that share the most similar bioclimate. This provides a more rigorous and natural method for identifying bioclimatic boundaries between vegetation types than the biomisation scheme, where thresholds are determined through more subjective expert-based approaches. Clustered areas may suggest bioclimate stability, while cluster edges are often regions of rapid bioclimate transition^19^. See “Clustering” in methods.

## Results

### Fragmented forests among connected savanna

Our modification of the biomisation scheme led to simulations of slightly less open central formation than Sato et al.^10^ (Fig. 1a), However, the same general patterns of reduced forest area and partial opening of the central Amazonia corridor remain in our pre-bias corrected results. Forest cover remains largely connected.

**Fig. 2:**
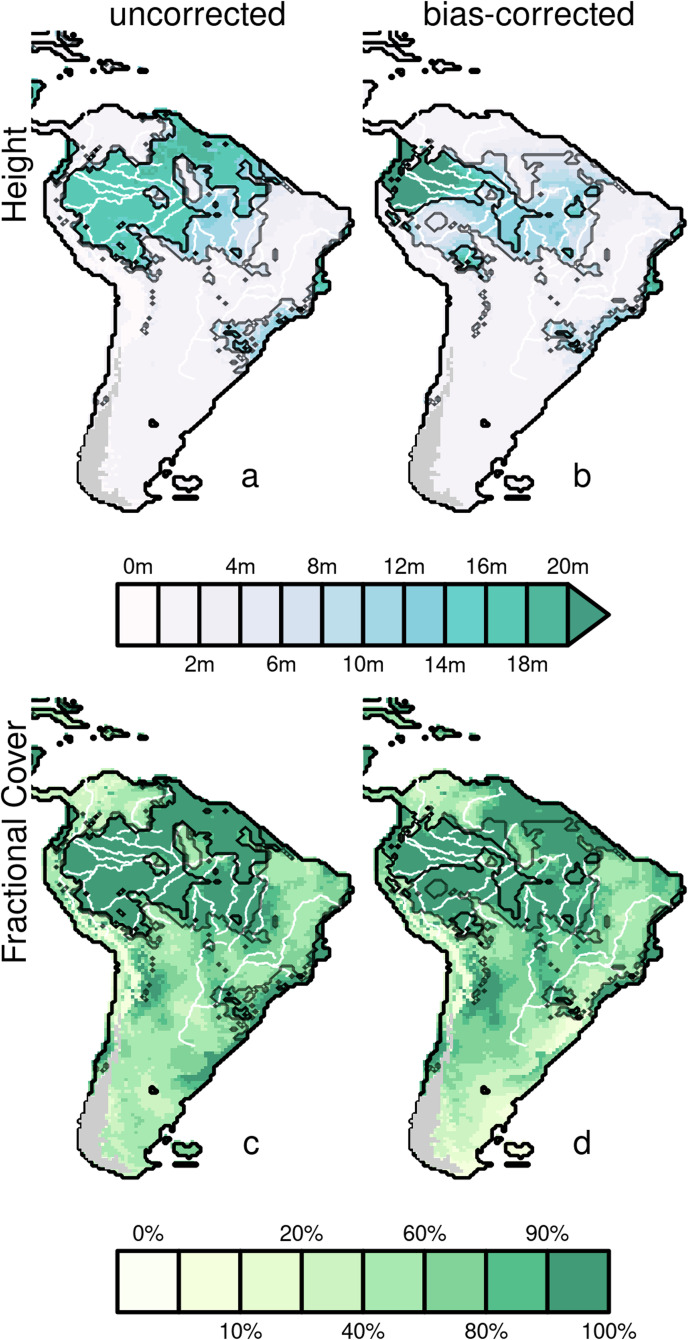
Height (top) and Fractional cover (bottom) before (left) after bias correction (right). Evergreen fraction, leaf type and growing degree days (GDD) are also bias-corrected but contribute less to changes in biome distribution (see Supplementary Figs. 2–7)

The Bias-corrected results for the ensemble reconstruction suggest a savanna-driven opening of the circum-Amazonian corridor along the Andes, the central Amazonian corridor, and the Northeast coastal corridor (Fig. 1b). Patterns of this simulated corridor suggest that the north and southeast’s open grasslands and short savannas tend to be connected by the more tree-dense, woodland/tall savanna (i.e., >60% vegetation cover with an average height between 5–10 m). However, simulated savannas of less than 5 m are still split into two major Northern (labelled ‘1’ in black in Fig. 2b) and Southern fragments, with Southern fragments almost divided by deserts into coastal (eastern present-day Caatinga; labelled ‘2a’ in Fig. 1b), central (Western present-day Cerrado labelled ‘2b’) and a Western pocket replacing present-day forest (‘2c’).

**Fig. 3:**
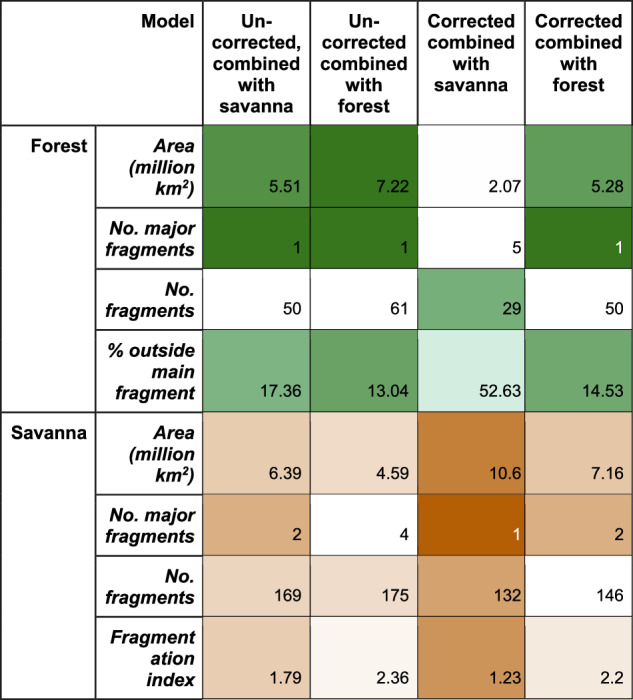
Forest and Savanna area, number of fragments and fragmentation index. Columns show uncorrected and bias-corrected simulations when considering “woodland /tall savanna” as part of the savanna (“combined with savanna”) or forest (“combined with forest”) biomes. We use shading to emphasise extent and connectivity (i.e. more shade for larger areas, few fragments/fragmentation).

Our results suggest that the Amazonian tropical humid forest may have split into at least 5 major fragments (Fig. 3). The largest fragment would have been in the moist western core of Amazonia (labelled ‘1’ in Fig. 1b), a second in central Amazonia (labelled ‘2’), and a third smaller fragment (‘3’) was located south along the Andes. Our ensemble reconstruction also shows 24 smaller forest fragments, with less than 10% of the area of the largest fragment, mostly in central and southern Amazonia and surrounded by woodland/tall savanna. As a comparable measure of fragmentation across biased and non-biased corrected reconstructions, 52.63% of forest area falls outside the main fragment in the bias-corrected results versus 17.36% in the uncorrected results (Fig. 3). Woodland/tall savanna compose much of the vegetation composition in the areas between forest and savanna and connects reconstructed tropical humid forest fragments. When combined with woodland/tall savanna, the number of major forest fragments reduces to 1 (Fig. 3). While there are more small forest fragments (49), these contribute much less to fragmentation, with just 17% of forest area outside the largest fragment (Figs. 1 and 3).

Our bias correction method will be more precise in the areas around pollen samples. We have less confidence in bias-corrected reconstructed vegetation in regions far from samples, particularly in the central Amazon (Fig. 1b), which shows the most savannifcation. However, a lack of data in humid regions introduces more noise rather than bias (Supplementary Fig. 8). While we focus on ensemble results, we also explore DVM outputs driven by individual climate models to sample model uncertainty in bioclimate variations in regions lacking pollen observations to mitigate some of the uncertainty introduced by the lack of pollen data. Most of the contributing reconstructions show similar general patterns. Savanna of less than 5 m in height may have been split between two major fragments in the north and south, with the southern showing signs of fragmentation from desert (forming two distinct fragments in reconstructions driven using HadGEM2 GCM model output), all connected by woodland/tall savanna except when driven by MIROC GCM, where a combination of eastern basin forest fragments and central amazon desert lead to two distinct major fragments. Fragmented forest is also similar across reconstructions, although HadGEM2 driven reconstruction suggests that continuous forest almost entirely constrained to the Western Basin may still be possible (Supplementary Table 2; Supplementary Fig. 9). All other reconstructions show a general pattern in the location of forest fragments, with the largest in the moist western core of Amazonia and a second to the east or southeast of that core (Supplementary Fig. 9); another third smaller fragment may have been located south along the Andes, with reconstructions driven using CNRM, FGOALS, and HADGEM GCMs showing the two smaller fragments in the ensemble’s southeastern Amazon reconstruction expanded and merged into a continuous fragment.

Bias correction affected all reconstructions, with all simulations showing substantially more savanna than their uncorrected counterparts (Fig. 1b vs. 1a, Supplementary Fig. 9) (5.51–7.06 vs. 2.07–3.54 million km^2^. Fig. 3; Supplementary Table 2), particularly in the Northeast of Amazonia. In all bias-corrected simulations, a savanna pathway resembling a central Amazonian dry corridor is present, unlike the uncorrected simulations where a dry central corridor only starts to form in two of five simulations (Supplementary Fig. 9) and does not fully open in the ensemble simulation (Fig. 1a). Grasslands/savannas merged into one major formation when connected by woodland/tall savanna (Fig. 1b), except for MIROC driven reconstructions, which forms two major fragments (Supplementary Fig. 9). Shorter formations (<5 m) by themselves are split between 2–4 major fragments (Fig. 1b, Fig. 3).

Height and FPC are the bias-corrected variables that contributed most to the savannafication inferred by our model reconstructions (Fig. 2). Bias-correction results in increased simulated canopy height in the western core of the Amazonian rainforest and widespread reductions in surrounding areas relative to the uncorrected reconstructions of Sato et al.^10^. The variations in height explain the formation of forest fragments and their boundary with woodland/tall savanna areas in our reconstructions. FPC undergoes less correction but alters the transition’s edges between forest/savanna and more arid biomes (Supplementary Fig. 9).

**Fig. 4:**
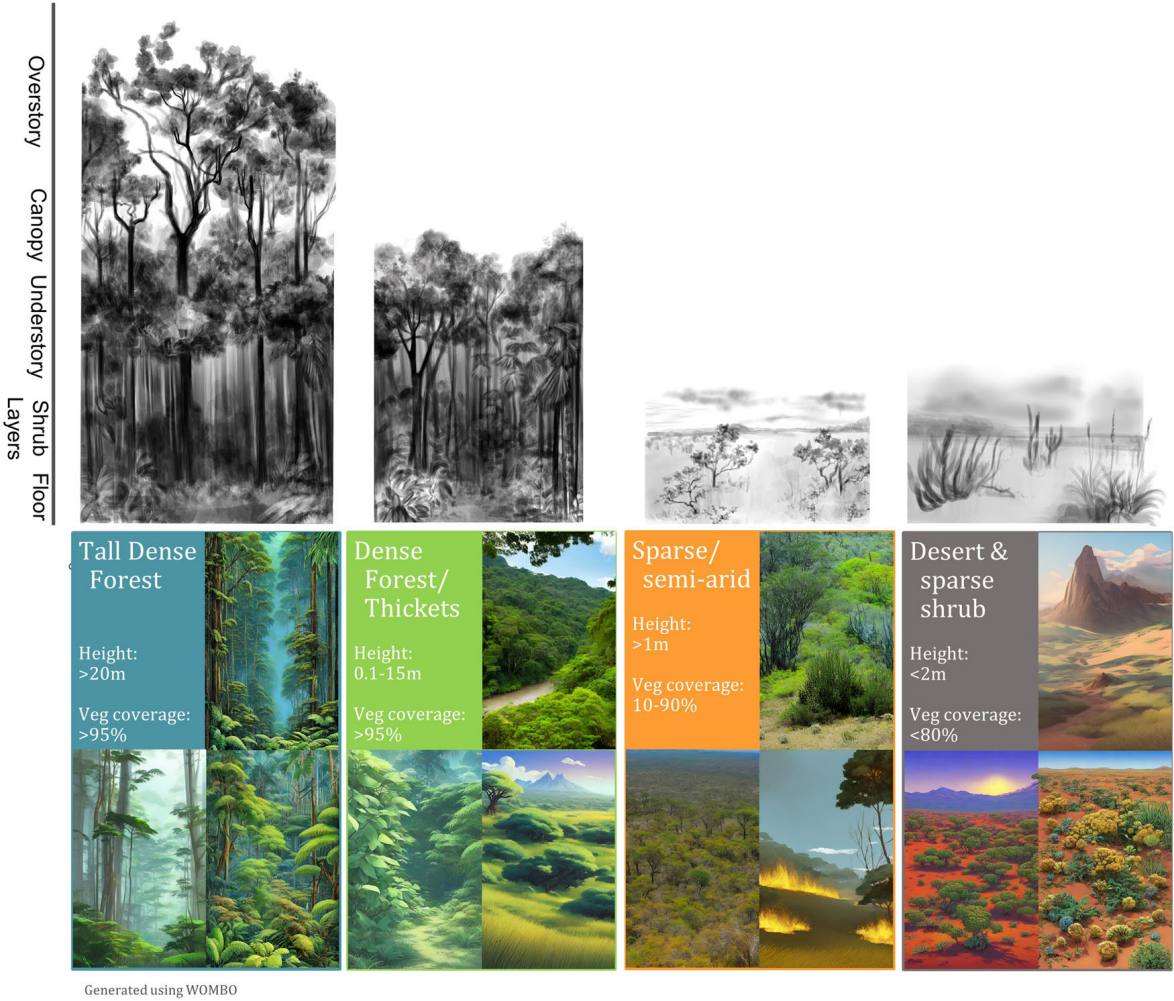
Possible images of clusters. Clusters are identified in Figs. 1c and f. Top-row artwork by Jennifer Lobo. Bottom row colour images were AI-generated. See “Vegetation assemblage imagery” in methods for generation.

In addition to the biomisation approach for defining areas of vegetation type, we also employ a machine-learning clustering approach (see Methods) to group data points into bio-climatically similar clusters (Fig. 1f). Using this technique, we identify vegetation types similar in height and FPC. Clustering identified four main regions in the ensemble run: Tall Dense forests, restricted to hilly areas in the North West (blue points, Fig. 1c, f); Dense forests and thickets with heights less than 20 m (green points, Fig. 1c, f); taller but sparse semi-arid vegetation with vegetation covers typically <90% (orange points, Fig. 1c, f); and short, sparse desert and shrubs (grey points, Fig. 1c, f). Given the broad definitions using just two variables, these vegetation types could span a range of ecosystems, possibly without modern-day analogous vegetation assemblages, displayed in Fig. 4, using artist and AI-generated images (see “Vegetation assemblage imagery” in methods). However, they provide a proxy of areas where species may have found spread easier, whilst cluster boundaries represent much larger bioclimatic differences that would more likely have impeded spread into different vegetation groups.

**Fig. 5:**
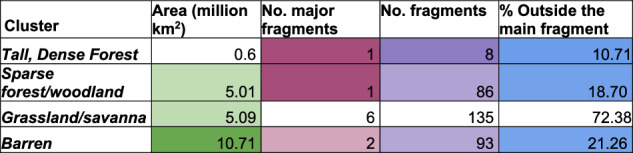
The area and fragmentation for biome clustering, as per Fig. 3. Bioclimatic clusters are identified in Fig. 1.

These clusters identify areas of taller, dense vegetation occupying a significantly different bioclimatic space than the more sparse vegetation, enabling generalists more adaptable to spread widely, but specialist species requiring niche conditions to remain more within small pockets. A connected cluster of dense vegetation with heights greater than ~1 m (Dense Forests/thickets) and taller (including Tall Dense Forests, Fig. 1c) formed across the Amazon. We found 18.7% of humid forests outside this main connected fragment (Fig. 5). The main fragment shows complex patterns with corridors and patches of more open vegetation running through (Fig. 1c) and 86 small fragments. This forest has corridors of less dense, savanna/grassland-like vegetation running up from the South (Fig. 1c) that is also more fragmented (6 major fragments and 72.38% outside the largest fragment). A mixture of forest and desert clusters causes this reconstructed fragmentation.

### Refugia Hypothesis

Our reconstructed forest refugia in Western Amazonia is close to Haffer’s predictions and the Inambari refugia to the south (Fig. 1b). There are also reconstructions of a forest patch with locations that overlap with the Rondônia refugia. Though we could interpret these isolated forest patches as refugia, the quantity and locations do not correspond precisely to Haffer’s hypothesised formations. However, given the uncertainties in climate simulations^20^, vegetation modelling^15^ and palynological reconstructions^11^, and uncertainties and qualitative nature of Haffer’s original reconstructions^1^, we would not expect exact correspondence.

Unlike Haffer’s Refugia of rainforest surrounded by open savanna, woodland/tall savanna connects the forest fragments in bias-corrected reconstruction. This ecotonal biome is composed of taller vegetation within savanna-like systems. This biome could correspond to areas of savanna characterised by denser, often deciduous (Supplementary Fig. 7) woody vegetation and is reflective of the heterogeneous nature of savanna vegetation. This woodland/tall savanna region connects disjoint forest fragments and disjoint savanna regions. Looking at clusters of similar vegetation coverage and height (Fig. 1c), there is also a small region of continuous closed forest extending into shorter, less-dense semi-arid areas to the southeast. Whether there are modern analogues to these past non-humid forests and woodland/tall savanna habitats is uncertain (see Fig. 4 for examples). However, the Cerrado gallery forests and transitional forests between Amazonia and the Caatinga^21^ may offer insight into the structure and fauna of these past ecosystems. Additionally, the broad distribution of white sand habitats, which have notably been found to have expanded in the past due to drier climate^22–24^ in the region corresponding to the northern central and coastal corridors (Fig. 1b,c), contributes additional complexity to the history of regional land cover^25^.

## Discussion

While our reconstructions show isolated forests and dense vegetation patches (Fig. 1b), they also suggest that woodland/savanna connected many with habitats of substantial height and canopy cover (Fig. 2) that could sustain both Amazonian and Cerrado species^4,21^. These may have also been areas of similar bioclimate, as identified in the separate clustering analysis, which revealed connected areas of both Sparse Forest and Savanna (Fig. 1c). The existence of these simulated habitats would have maintained the connectivity and gene flow among isolated forest patches without major barriers, as is observed in the current absence of genetic structure of animals and plants within interfluvials^26–29^. The expansion of woodland/savanna and the more open savannas could also explain the lack of genetic breaks among populations of savanna animal species within and across Amazonia^30–33^. The response of less vagile species could be one of the occasional dispersals facilitated by the expansion of savanna-like habitats and diversification after they recede^34^, and vice-versa in the case of forest species^35^. Conversely, low-dispersal savanna plants can become isolated, leading to population structure among isolated Amazonian open vegetation relicts and the Cerrado^36–38^, implying that the woodland/tall savannas habitats could be a depauperate community composed of species of Amazonian origin rather than savanna.

Our inferences here depend on fusing proxy reconstructions of vegetation with model output, but there are few proxies for today’s central Amazon. There is agreement across our five reconstructions, which gives us confidence in the spatial distribution of forests. However, more proxy data from central Amazonia for the LGM would help refine these results in the future. Our results also depend on the pre-defined bioclimatic thresholds between forest and savanna, and there is the potential for our vegetation reconstruction and bias correction to change with the choice of thresholds used in the biomisation scheme. Although testing a range of thresholds suggests that our biome distributions and fragmentation level (Supplementary Fig. 10) are largely insensitive to the selection of thresholds. See methods for tests and explanation. However, a less subjective data-driven definition of biome differences, such as those we started introducing when clustering bioclimates, would help extend our technique to explore variation in bioclimate in more detail and how this might affect specific species distribution.

Ensemble reconstructions of the Atlantic Forest (Fig. 1c) are also consistent with previous studies that show stable northern, central, and southern refugia in the region^39^, connected by a gradient of shorter and open canopy forests to open vegetation habitats. These areas between refugia match boundaries where phylogenetic turnover occurs for many organisms, including birds, amphibians, butterflies, and plants^40^, while populations from refugia areas usually present higher genetic diversity^39^. This phylogeographic evidence is generally consistent with our bias-corrected model reconstructions, showing how the dynamics of past vegetation reduced gene flow and shaped current genetic diversity patterns in the Atlantic Forests. Our results also show that putative connections between the Atlantic Forest and Amazonia could have occurred through the three proposed corridors in North-eastern, Central, and Southern Brazil^12^ in the Late Pleistocene.

We present a novel picture of glacial vegetation in the South American tropics with essential consequences for dispersal and diversification that is more complex than its predecessors. Our results were derived through bias-correction of model reconstructions against empirical pollen data, providing a rare example of spatial integration of model and palaeoecological data. Our bias-corrected reconstructions are the first quantitative syntheses of DVM output and palynological records. It suggests a degree of savannafication even greater than those of previous studies^10^. Bias correction resulted in a consistent reconstruction of a central Amazonian corridor connecting northern and eastern open biomes. Without bias corrections, the Amazonian rainforest experienced dieback in its borders, sometimes to a large degree with intrusion into internal areas, but mainly remained connected as a single unit. With bias correction, Amazonian rainforests were split into distinct fragments.

Unlike Haffer’s well-defined forest refugia and savanna barriers, our bias-corrected reconstructions suggest a complex mosaic of open, semi-open, and closed habitats. There were signs of stable, moist forests with potential geographical correspondence to Haffer’s refugia but connected by a woodland/tall savanna and complex webs of dense vegetation. We posit that this ecotonal biome could be a barrier for certain specialist species and a corridor for more vagile generalists. This species-specific filter would then form ‘semi-refugia’, relaxing some of the conditions of Haffer’s initial hypothesis. However, our results also suggest that past Amazonian environments are complex and nuanced in mediating dispersal, and it may be time to advance beyond notions of simple forest refugia.

We show that as Earth System modelling techniques advance, more reliable and detailed reconstructions of past vegetation will become available for interpretation in biogeographical contexts. Conversely, pursuing compelling questions, such as the unknown origins of Amazonian biodiversity, can effectively motivate and guide modelling studies.

## Methods

### LPX vegetation model

We used Land surface Processes and eXchanges (LPX) model^13^ simulations and pollen-based reconstructions of the LGM as described in Sato et al. and Calvo et al.^10,15^. These studies drove LPX with paleoclimate simulations from four GCMs^41,42^ to produce four vegetation reconstructions of South America during the LGM. We use a fifth reconstruction, assembled by Sato et al., who took the average of each bioclimatic output from the four reconstructions. We used Sato et al.’s^10^ simulations with active fire representation and low CO_2_ impacts.

LPX fire has been evaluated against paleo data and present-day vegetation cover and height observations^10,13,15,43,17^, and its CO_2_ fertilisation response reproduces the magnitude of the land carbon sink^44^ identified by the Global Carbon Project^45^. LPX’s present-day fire and vegetation simulations have undergone extensive benchmarking to demonstrate its ability to reproduce observed spatial patterns and trends in vegetation cover, fire and CO_2_ response^13,43,44,46–48^. The LGM climate simulations we used also compare well against paleo-proxy reconstructions of sea surface temperature proxies^49^. Martin Calvo et al^15^. Sato et al.^10^ compared the LPX outputs we used in this study to global and South American pollen vegetation reconstructions, showing the modelling framework had skill in reproducing LGM vegetation distributions.

Here, we perform additional benchmarking to quantify this skill by comparing our biome reconstructions against our pollen observations using the Discrete Manhattan Metric (DMM) described in Sato et al. DMM is the mean of the distance between the simulated and observed biome across all pollen sites. The closer the biomes are to a given site, the smaller the contributed score, by the mean difference between biomes in Supplementary Table 1. So, for example, if simulations and observations agree at a site, then that site contributes a score of 0. If the biomes are as opposite as possible, it contributes a value of 1. An overall score of 1 represents complete disagreement, whereas 0 represents complete agreement. The ensemble model returns the best score of 0.117, followed by FGOALS with 0.134; HadGEM with 0.141; CNRM with 0.157; and MIROC with 0.159. All scores are better than two null models: if a simulation gave tropical forests across the continent (0.235) and savannas across the continent (0.229). Single-value null models are becoming a standard for DVM model benchmarking^50–52^. Scores are proportional to the distance from observations and, therefore, performance, so our ensemble model is a 49% improvement on the best single-value null model. In contrast, individual models range from 32-41% improvement.

### Biomisation

Our biomisation scheme (Supplementary Fig. 1) converted variables into biomes (Fig. 1). This scheme adapts the previous LPX papers^10,15,16^ to formalise the description of “dominant” PFTs. Prentice et al.^16^ and Ciais et al.^17^ devised the schemes thresholds to translate LPX output to biomes for data–model comparisons. These studies calibrated thresholds that best translated model output to present-day biome distributions. Note that neither study tested South American vegetation distributions at the LGM, and thresholds were, therefore, chosen independently of the hypothesis we are testing. Many studies subsequently used these thresholds^10,15,53–55^.

We slightly updated the biomisation scheme to quantitatively compare six variables between pollen and models to perform the bias correction. The update tests evergreen vs deciduous and tropical vs temperate vs tropical ecosystems, so it has little influence on our forest vs open biome types test. We also introduced a new ecotone type to test the transition between forest and savanna: woodland/tall savanna, which follows the same definition as tropical savanna but has an average height of 5–10 m.

### Bias-correction

We translated the pollen-based biomes into bioclimatic ranges of six model output variables (Supplementary Table 1): total foliage projection cover (FPC); evergreen fraction (EG); tropical fraction (TR); temperate fraction (TM); height (H); and growing degree days (GDD). Thresholds were the same as used in the biomisation scheme. We bias-corrected model output across all these variables against these pollen sites. Model output and pollen-based observed ranges were transformed per Supplementary Table 1 so that data were approximately normally distributed: logit for fractional variables and log for GDD with [0, ∞) bounds. A height of 130 m appears to be the hydrologically limiting height of trees^56^, which we set as a height limit for biomes and use a logit transformation to height over 130 m. We interpolated the difference between the transformed LPX output and the extreme range for each pollen site by fitting a thin plate spline surface using Tps with default settings in the “fields” r package^57^ in R3.6.2^58^. We subtracted these surfaces for each bioclimate variable from LPX output (Supplementary Figs. 2–7).

Linking the thresholds used in bias correction to biomisation helps make our results less sensitive to the choice of thresholds. LPX output provides variations in vegetation between pollen points, and scaling the thresholds simply scales the LPX output during bias correction. To demonstrate this, we performed two sensitivity tests (Supplementary Fig. 9):

FPC threshold between Desert/grass/savanna and forest changes to 0.2 and 0.4. Height threshold between savanna/tall savanna/forest changes to 2.5 and 5.

FPC threshold between Desert/grass/savanna and forest changes to 0.5 and 0.75. Height threshold between savanna/tall savanna/forest changes to 10 and 20.

We chose FPC and height as these were the variables that most controlled the distributions of the vegetation types we were interested in.

### Clustering

The biomeisation scheme intersects dense areas of bioclimatic space (Fig. 1 d, e). We use a k-means clustering approach in Fig. 1 c, f to identify these areas of common bioclimates in a more objective way. K-means clustering is a data analysis technique used to group similar data points into clusters based on their proximity. An unsupervised machine learning algorithm aims to partition a dataset into distinct bioclimatic regions. We clustered bias-corrected results using k-means in R3.6.2^58^ on height and FPC variables, following^19^.

### Vegetation assemblage imagery

In Fig. 4, we use AI-generated images of possible vegetation composition rather than photos to capture that, during the LGM, vegetation composition and structure of ecosystems may have been different from today. Images for each clusters were generated by converting the cluster bounds into text and including descriptions of South American landscapes and common genus into Wombo Large Language Model LLM AI image generator (https://dream.ai/create). We used the following descriptions:

- Tall Dense Forests: “Tall Dense Forests with vegetation with average height above 20 m covering more than 95% of the area. Amazon forest tree and plant species”
- Dense Forests/thickets: “Dense Forests, woodland and thickets with vegetation between 0.1 and 20 m covering more than 95% of the area. Amazonia and Cerrado species. Mosaics between dense forests and thickets and dryer gallery-like forests.”
- Sparse savanna/grass: “Woodland, savanna and grassland with vegetation above 1 m, sometimes much taller, covering between 50-95% of the land. Up to 50% short grass or bare soil. Caatinga and Cerrado species, including Handroanthus and cacti. Seasonal vegetation including fire”.
- Sparse shrub: “Shrub, grass and desert. Vegetation is mostly less than 1 m, sometimes taller, covering 0-95% of the land. Up to 90% short grass or bare soil. South American desert species.”

These images were filtered by several South American Ecologists and climate experts (see acknowledgements) for physically implacable characteristics until we had three images for each cluster. We excluded images for these reasons:

- Contained people or vehicles.
- Looks too like autumn in temperate regions.
- Too reminiscent of Cape and Nama Karoo Floristic region endemic in South Africa.

### Fragmentation assessment

We found the number of forest or savanna fragments (Fig. 3, Supplementary Table 2) by converting a map of each biome in turn vs other cover types for contiguous Southern America to polygons using rasterToPolygons from the raster package^59^ in R3.6.2. The number of major fragments is the number of biomes polygons with an area greater than or equal to 10% of the largest polygon. “Total fragments” is the number of polygons, while the “% outside the main fragment” is the percentage of the total biome area outside the largest polygon.

## Supplementary Information

Supplementary Information

## Data Availability

Pre- and post-bias corrected data that support our findings are available at the Zenodo repository 10.5281/zenodo.7716010^60^.

## Code availability

The code for the version of LPX used in this study is available at the Zenodo repository (10.5281/zenodo.4757522)^13,61^. Bias-correction code is freely available on github and will be posted on zenodo following review: https://github.com/douglask3/LPX_equil/tree/be7efbe6bc974c4c9f5b65e7282971a089714359.
