# Supplementary material for: Niche-dependent forest and savanna fragmentation in Tropical South America during the Last Glacial Maximum
Source: NPJ Biodivers. 2024 Sep 11;3:23. doi: 10.1038/s44185-024-00056-4 (PMC11391077; doi:10.1038/s44185-024-00056-4)
Supplement: Supplementary file 1 — Supplementary Information [file 44185_2024_56_MOESM1_ESM.pdf]

Supplementary Information

Niche-dependent forest and savanna fragmentation in Tropical South America during the Last Glacial Maximum

*Douglas I Kelley[1, *], Hiromitsu Sato[2, *], Michaela Ecker[3], Chantelle Burton[4], Joao M G Capurucho[5], John Bates[6]*

[1] UK Centre for Ecology & Hydrology, Wallingford, Oxfordshire, OX10 8BB, UK

[2] Department of Earth Sciences, University of Toronto, Ontario, Canada

[3] Institute of Pre- and Protohistoric Archaeology, Kiel University, 24118 Kiel, Germany

[4] Met Office, Fitzroy Road, Exeter. EX1 3PB UK

[5] Coordenação de Biodiversidade, Instituto Nacional de Pesquisas da Amazônia (INPA), Manaus, Av. André Araújo, 2936, AM, Brazil.

[6] Integrative Research Center, Field Museum of Natural History, Chicago, IL, USA

[*]Correspondence:

Douglas I Kelley: doukel@ceh.ac.uk

Hiromitsu Sato: satohiro86@gmail.com

Supplementary Table 1. Bioclimatic limits and transformation information for biomisation scheme and bias correction.

**Biome**	**FPC (fraction)**		**EG (fraction)**		**TR (fraction)**		**TM (fraction)**		**Height (m)**		**GDD (℃)**	
	min	***max***	***min***	***max***	***min***	***max***	***min***	***max***	***min***	***max***	***min***	***max***
Trans-formation	logit		logit		logit		logit		logit(x/130)		log	
Inverse trans-formation	logistic		logistic		logistic		logistic		130× logistic(x)		exp	
Tropical Humid Forest	0.6	1.0	0.5	1.0	0.5	1.0	0.0	0.5	10	N/A	350	N/A
Woodland /tall Savanna	0.6	1.0	0.0	1.0	0.5	1.0	0.0	0.5	5	10	350	N/A
Tropical Dry Forest	0.6	1.0	0.0	0.5	0.5	1.0	0.0	0.5	10	N/A	350	N/A
Warm Temperate Forest	0.6	1.0	0.5	1.0	0.0	0.5	0.5	1.0	10	N/A	350	N/A
Temperate Deciduous Forest	0.6	1.0	0.0	0.5	0.0	0.5	0.5	1.0	10	N/A	350	N/A
Tropical Savanna	0.6	1.0	0.0	1.0	0.5	1.0	0.0	0.5	0	10	350	N/A
Sclerophyll Woodland	0.6	1.0	0.0	1.0	0.0	0.5	0.5	1.0	0	10	350	N/A
Temperate Parkland	0.6	1.0	0.0	1.0	0.0	0.5	0.5	1.0	0	10	350	N/A
Boreal Parkland	0.6	1.0	0.0	1.0	0.0	0.0	0.0	0.5	0	10	0	350
Dry Grass/ Shrub	0.3	0.6	0.0	1.0	0.0	1.0	0.0	1.0	0	N/A	350	N/A
Hot Desert	0.0	0.3	0.0	1.0	0.0	1.0	0.0	1.0	0	N/A	350	N/A
Shrub Tundra	0.3	0.6	0.0	1.0	0.0	0.0	0.0	0.5	0	N/A	0	350
Tundra	0.0	0.3	0.0	1.0	0.0	0.0	0.0	0.5	0	N/A	0	350

Supplementary Table 2. Summary of fragmentation for bias-corrected model output. Forest and Savanna area, number of fragments and fragmentation index. See Table 1 caption.

	Model	CNRM	FGOALS	HadGEM	MIROC	Ensemble		
"woodland/tall savanna" as part of the savanna biome								
**Forest**	***Area (million km^2)***	3.054	2.954	2.4	2.14	2.07		
	No. major fragments	5	3	1	4	5		
	No. fragments	34	24	29	31	29		
	% outside main fragment	58.16	51.22	25.37	50	47.37		
**Savanna**	***Area (million km^2)***	9.63	9.56	8.38	11.83	10.6		
	No. major fragments	1	1	1	2	1		
	No. fragments	136	110	119	139	132		
	% outside main fragment	20.63	17.36	18.7	41.18	18.7		
"woodland/tall savanna" as part of the forest biome								
**Forest**	***Area (million km^2)***	5.23	4.79	6.3	3.69	5.28		
	No. major fragments	1	1	2	1	1		
	No. fragments	37	25	37	33	50		
	% outside main fragment	12.28	9.09	16.67	25.37	14.53		
**Savanna**	***Area (million km^2)***	7.34	7.61	4.41	10.11	7.16		
	No. major fragments	2	2	3	2	2		
	No. fragments	136	118	115	141	146		
	% outside main fragment	59.51	59.51	59.51	59.51	59.51	45.65	56.

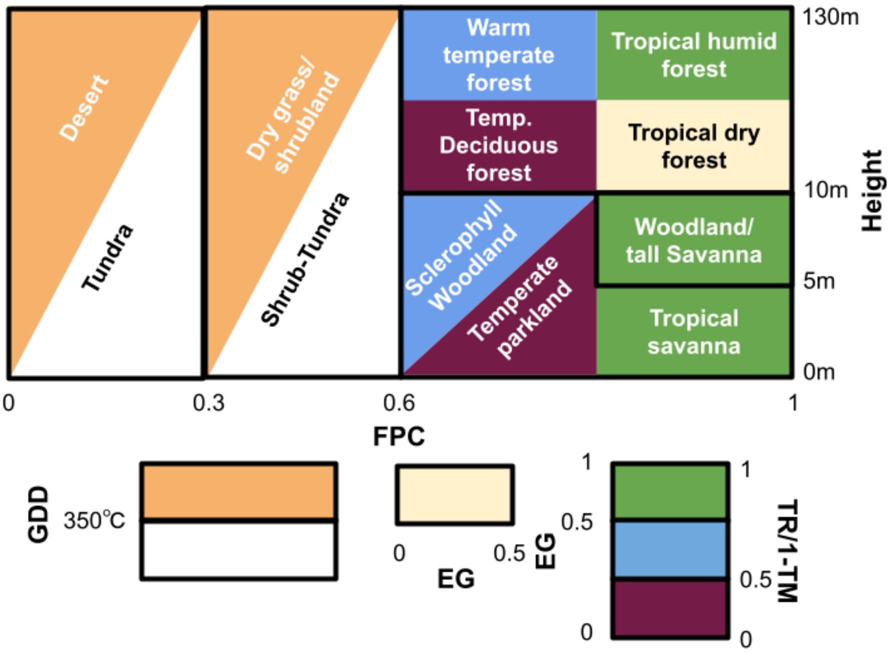

Supplementary Figure 1. Biomisation scheme. We primarily split biomes by Fraction Projected Covers (FPCs) of 0.3 and 0.6, with biomes > 0.6 split by a height of 10m. Forests (>0.6 FPC and > 10m) is split by Growing Degree Days (GDD), Evergreen FPC (EG) and Tropical or temperate FPC (TR, TM). Likewise, we split FPCs> 0.6 and heights <10m into savanna, woodland and parkland using EG and TR. We additionally assign Tropical savanna >5m to Woodland/Tropical savanna. We divided desert, dry grassland and (shrub)-tundra by FPC of 0.3 and GDD of 350°C.

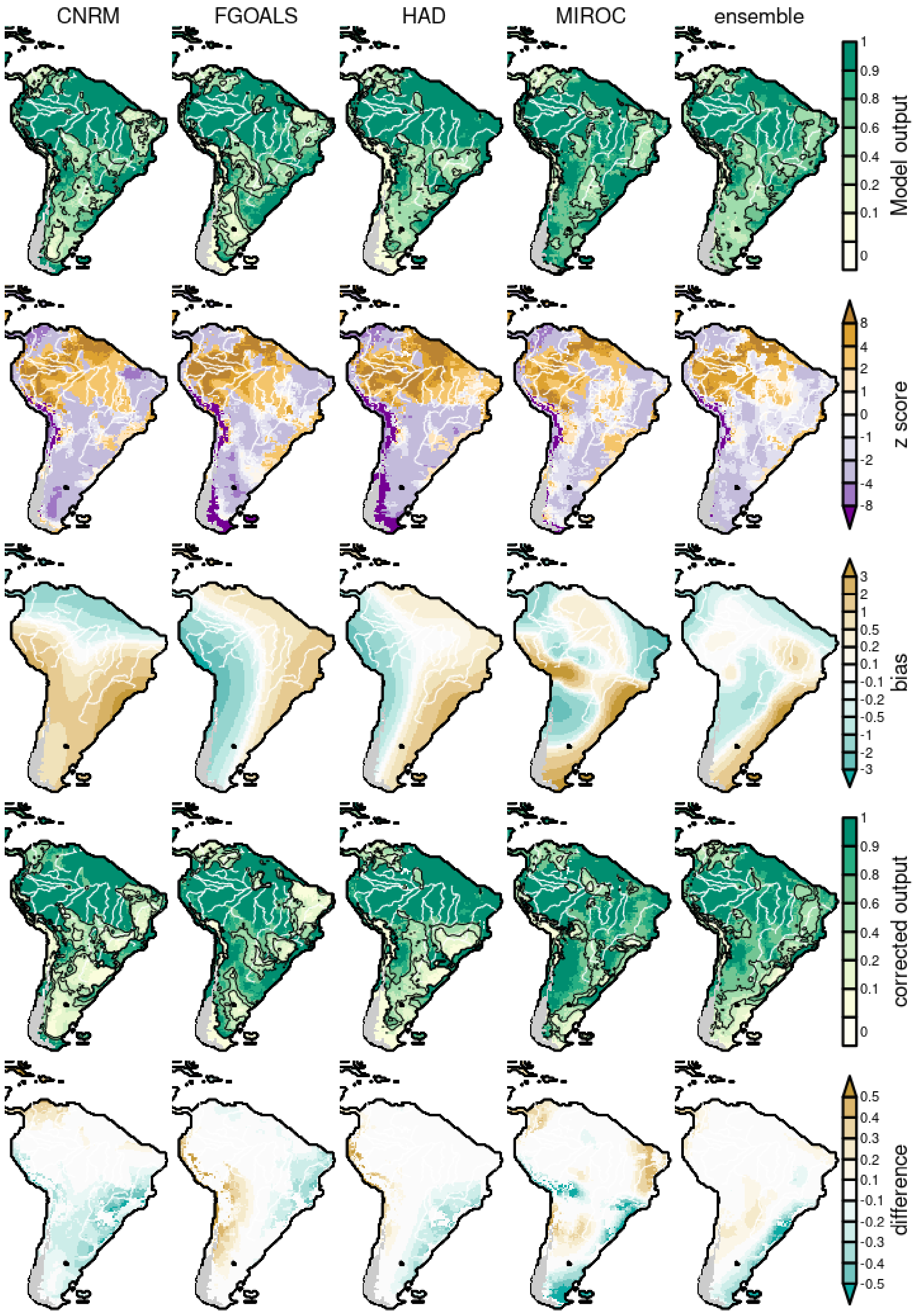

Supplementary Figure 2. Total vegetated Fractional Projected Cover (FPC) bias correction. The top row shows LPX outputs of FPC. The 2nd row shows FPC cover converted to z-scores. Based on the interpolated distance between these and pollen scores, we apply a correction (3rd row) to produce the corrected FPC (4th row). The 5th row is the difference between pre and post-corrected FPC. Contours for FPC values (1st and 4th row) show biome scheme boundaries at 0.3 and 0.6 (Supplementary Figure 8).

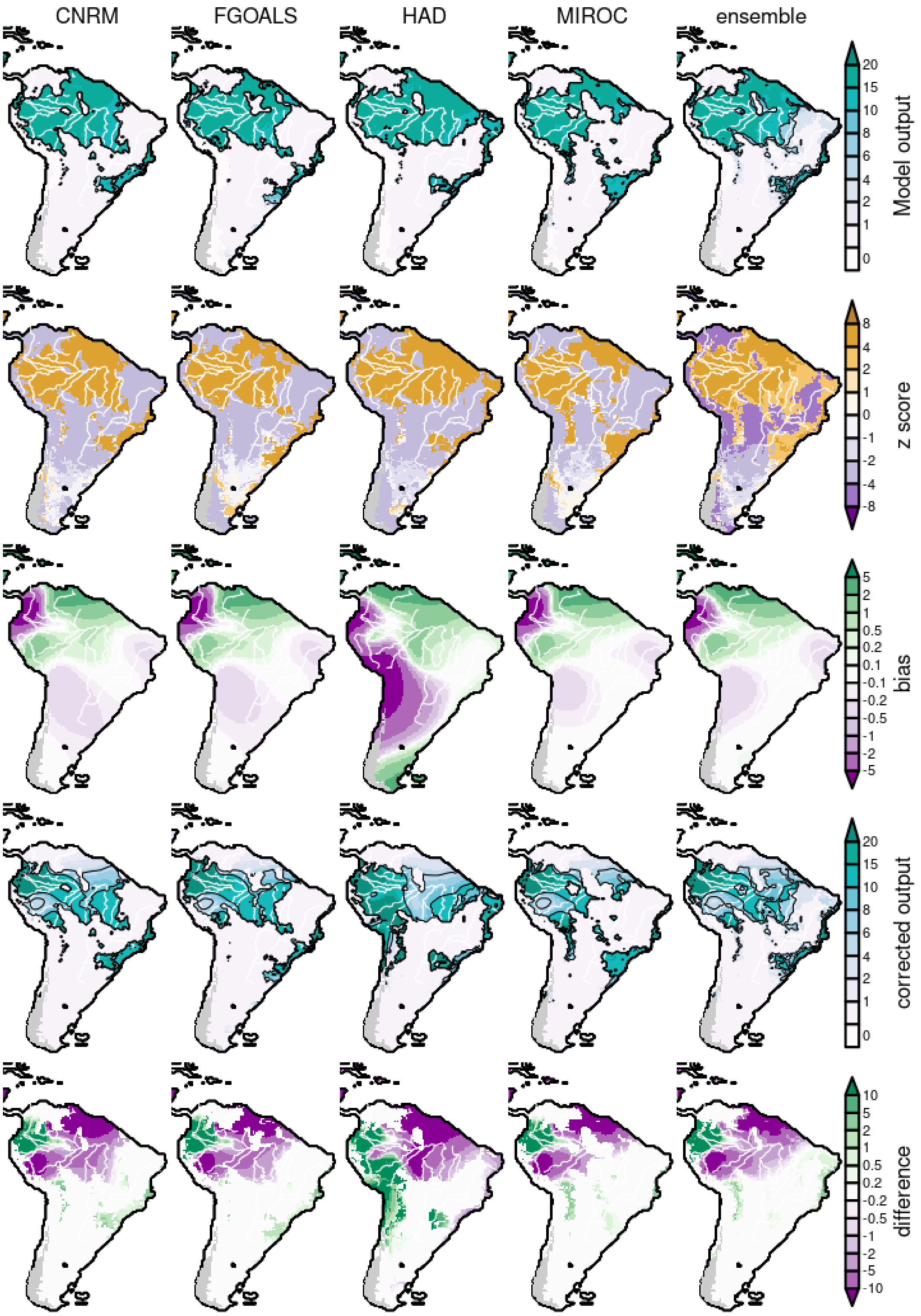

Supplementary Figure 3. height bias correction. As per Supplementary Figure 1 but for height in meters. Contours for height values (1st and 4th row) show biome scheme boundaries at 5m and 10m (Supplementary Figure 8).

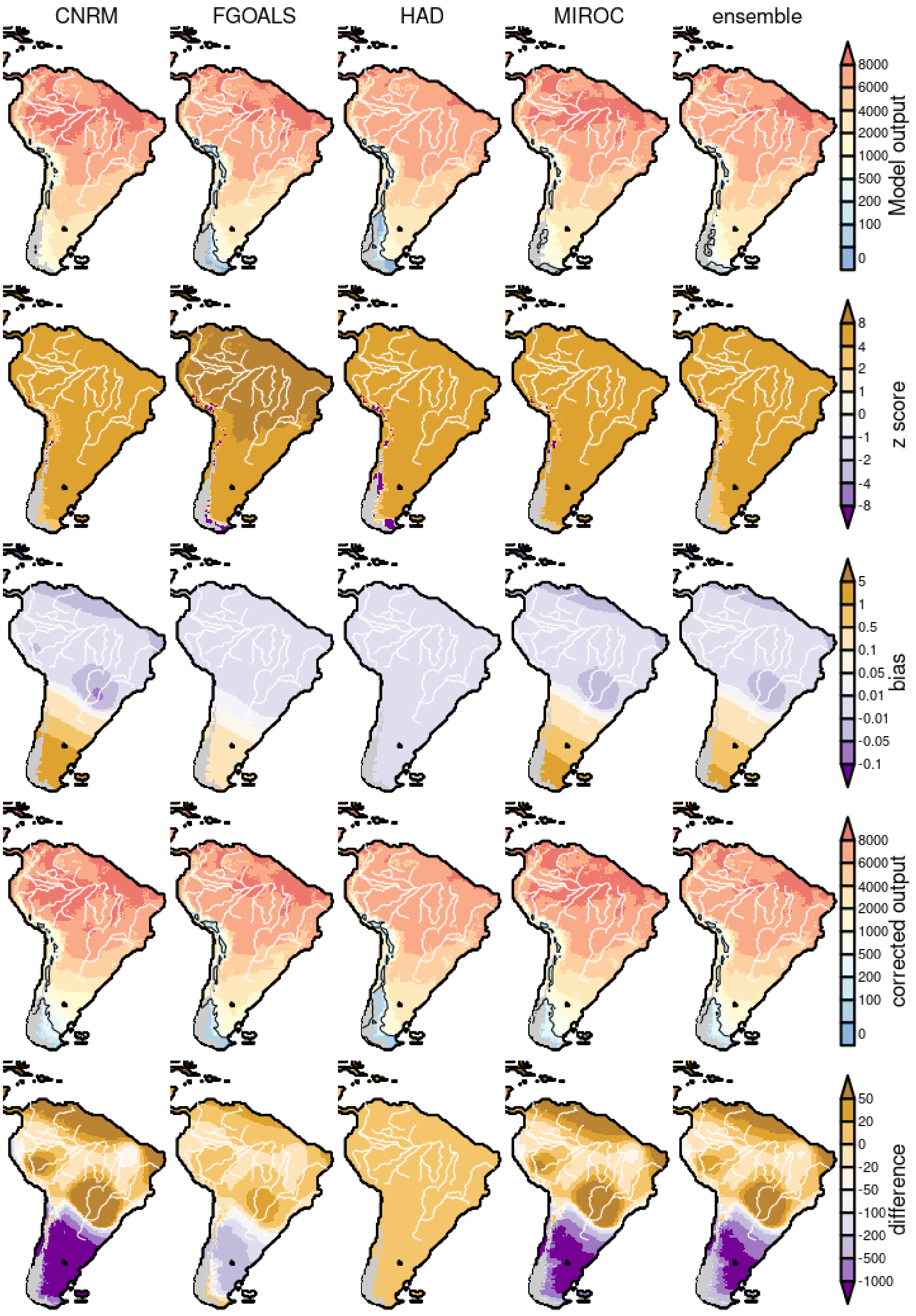

Supplementary Figure 4. Growing Degree Days (GDD) bias correction, As per Supplementary Figure 1 but for GDD in °C. Contours for GDD values (1st and 4th row) show biome scheme boundaries at 350℃ (Supplementary Figure 8).

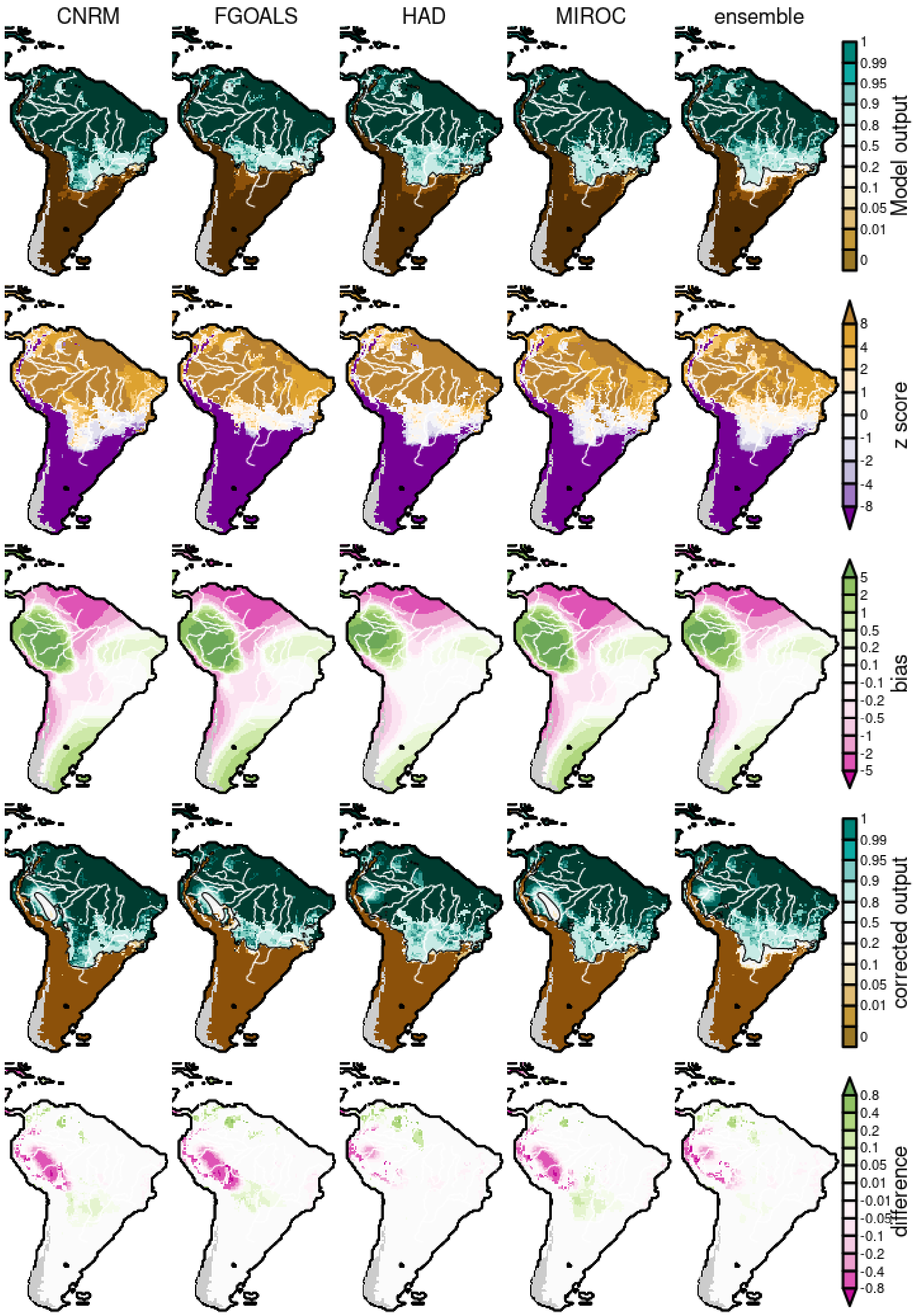

Supplementary Figure 5. Tropical FPC bias correction. As Supplementary Figure 1 but for just tropical vegetation (LPX plant functional types of Tropical Boradlead evergreen/raingreen tree and C4 grasses, see [14]). Contours for cover values (1st and 4th row) show biome scheme boundaries at 0.5 (Supplementary Figure 8).

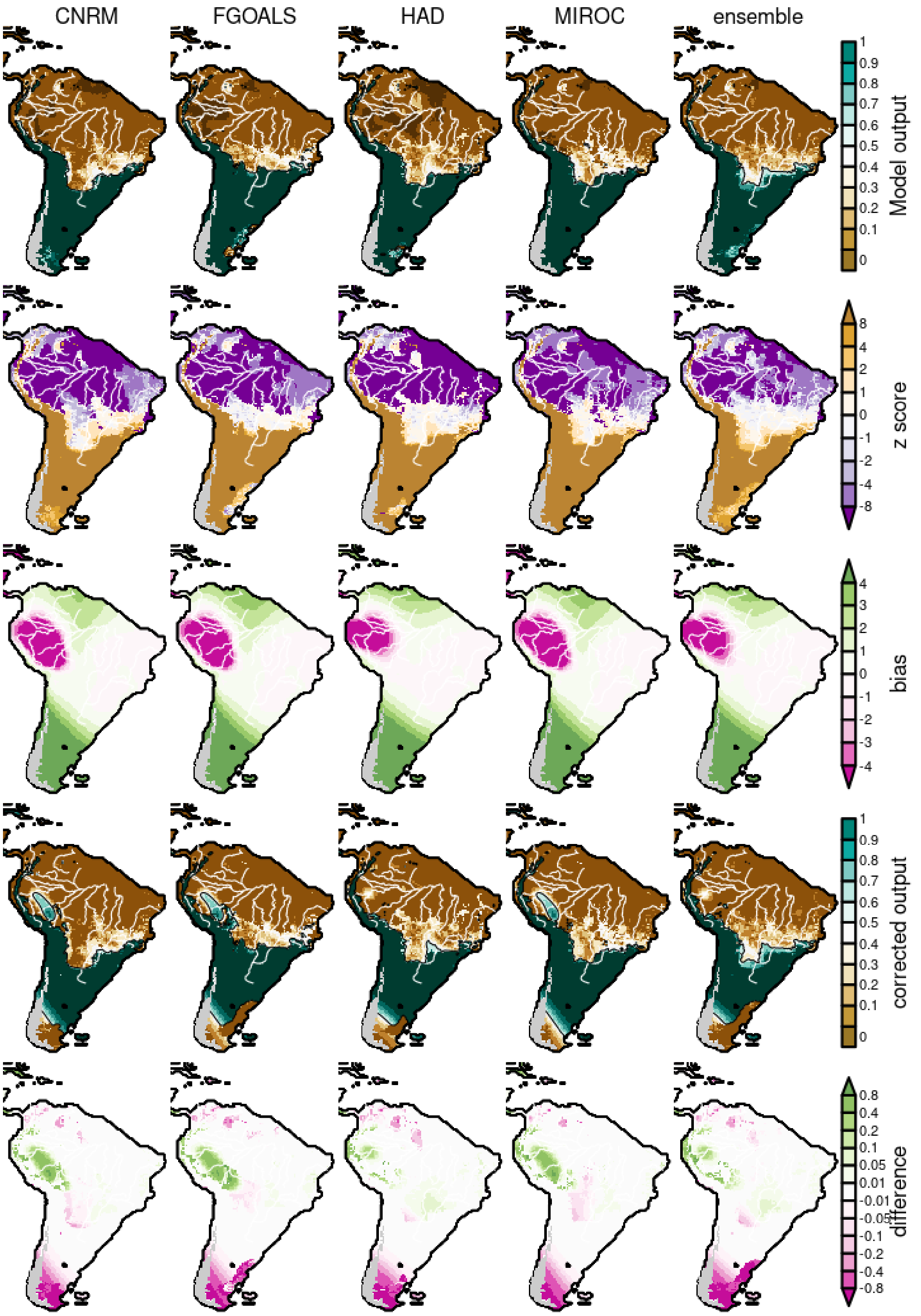

Supplementary Figure 6. Temperate FPC bias correction. As per Supplementary Figure 1 but for just temperate vegetation (LPX plant functional types of Temperate Broadleaf evergreen/summergreen and temperate needleleaf trees and C3 grasses, see [14]). Contours for cover values (1st and 4th row) show biome scheme boundaries at 0.5 (Supplementary Figure 8).

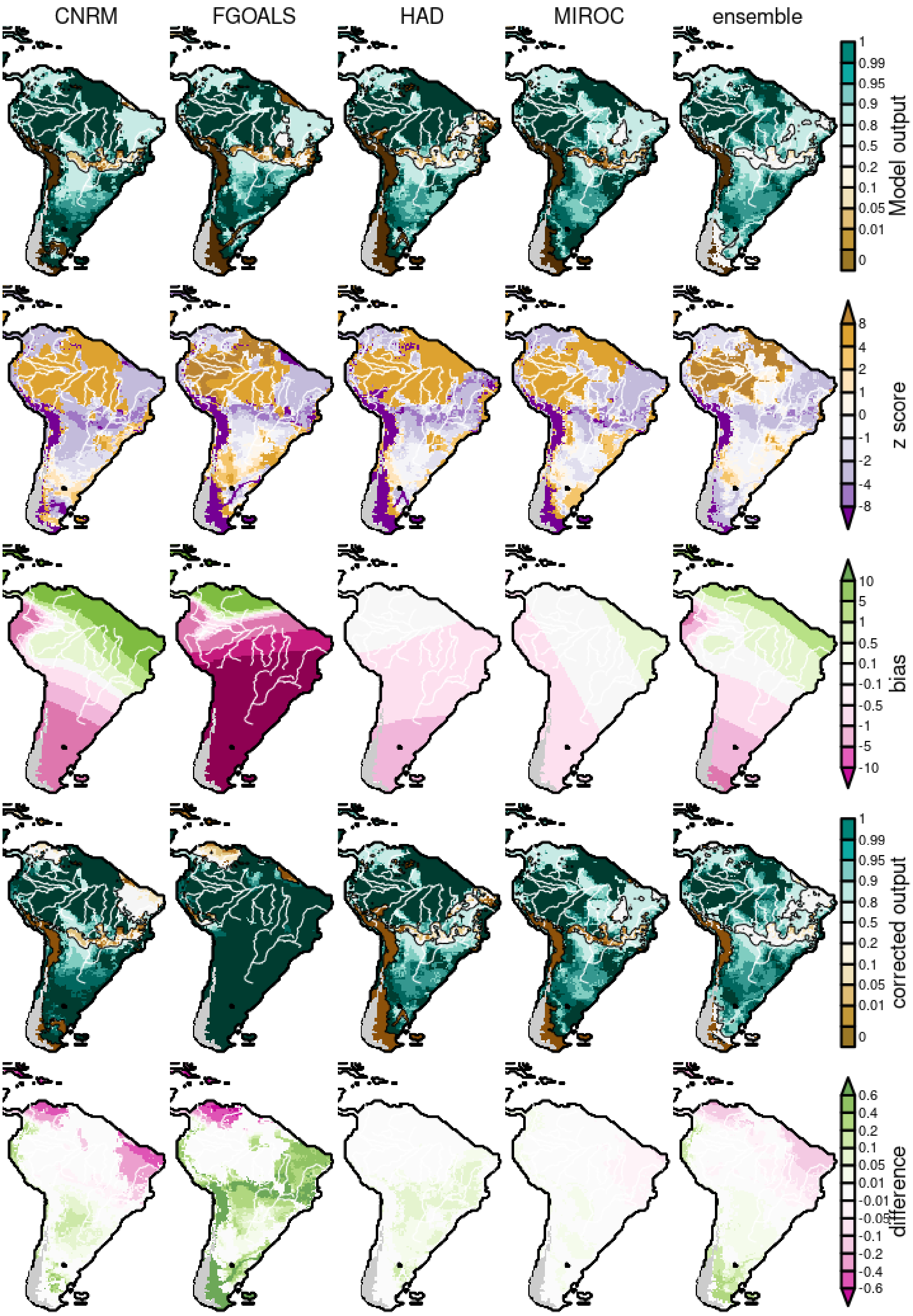

Supplementary Figure 7. Evergreen FPC bias correction. As per Supplementary Figure 1 but for just evergreen vegetation (LPX plant functional types of Temperate Broadleaf evergreen and temperate broadleaf evergreen, and needleaf evergreen trees as a fraction of all trees, see [14]). Contours for cover values (1st and 4th row) show biome scheme boundaries at 0.5 (Supplementary Figure 1).

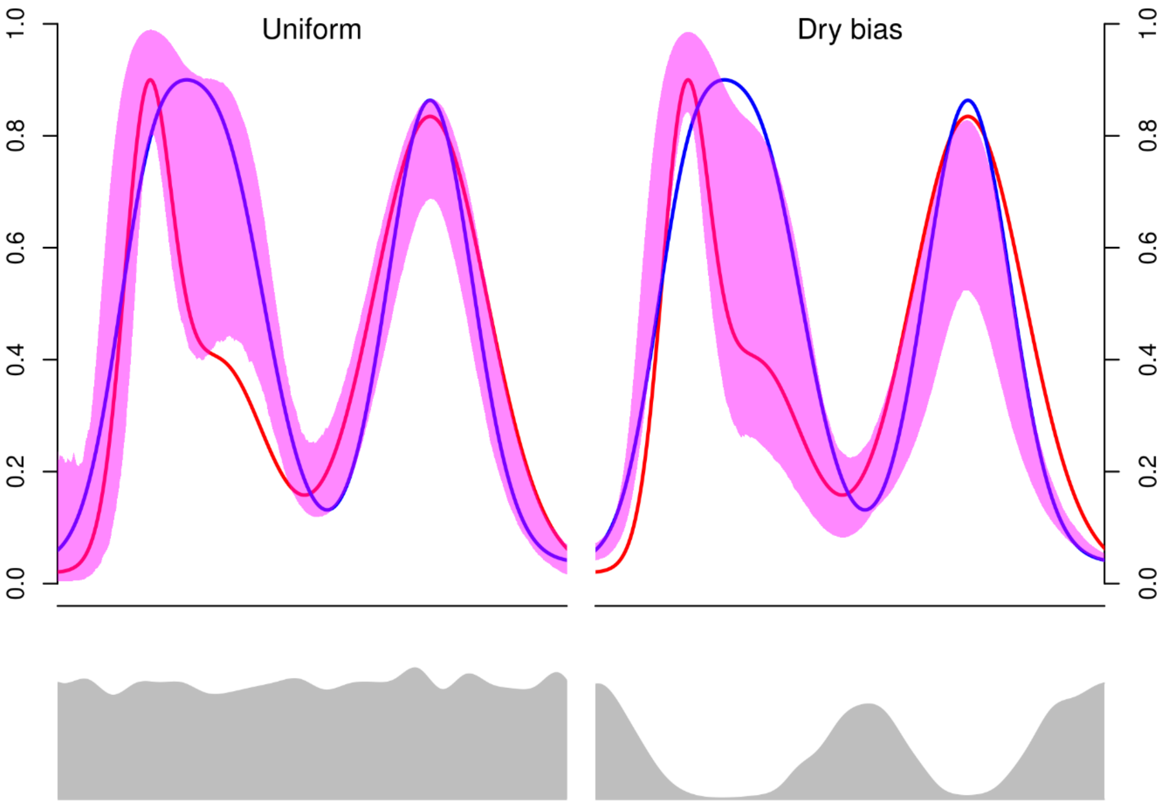

Supplementary Figure 8. A demonstration of bias-correction for FPC (y-axis) over 1-dimensional transect (x-axis). The red line represents model output, and blue a possible true state. The purple represents the 5-95% quantile of 1000 bias-corrections, with each bias-corrected to 10 randomly sampled points (equivalent to pollen sites) on the blue “true state” line. The grey histogram is the distribution of the (1000 x 10) 10,000 randomly-sampled sample point locations. On the left shows bias-correction if there was no sampling point location bias. The right shows if sampled points have a tendency to fall in areas of low actual FPC.

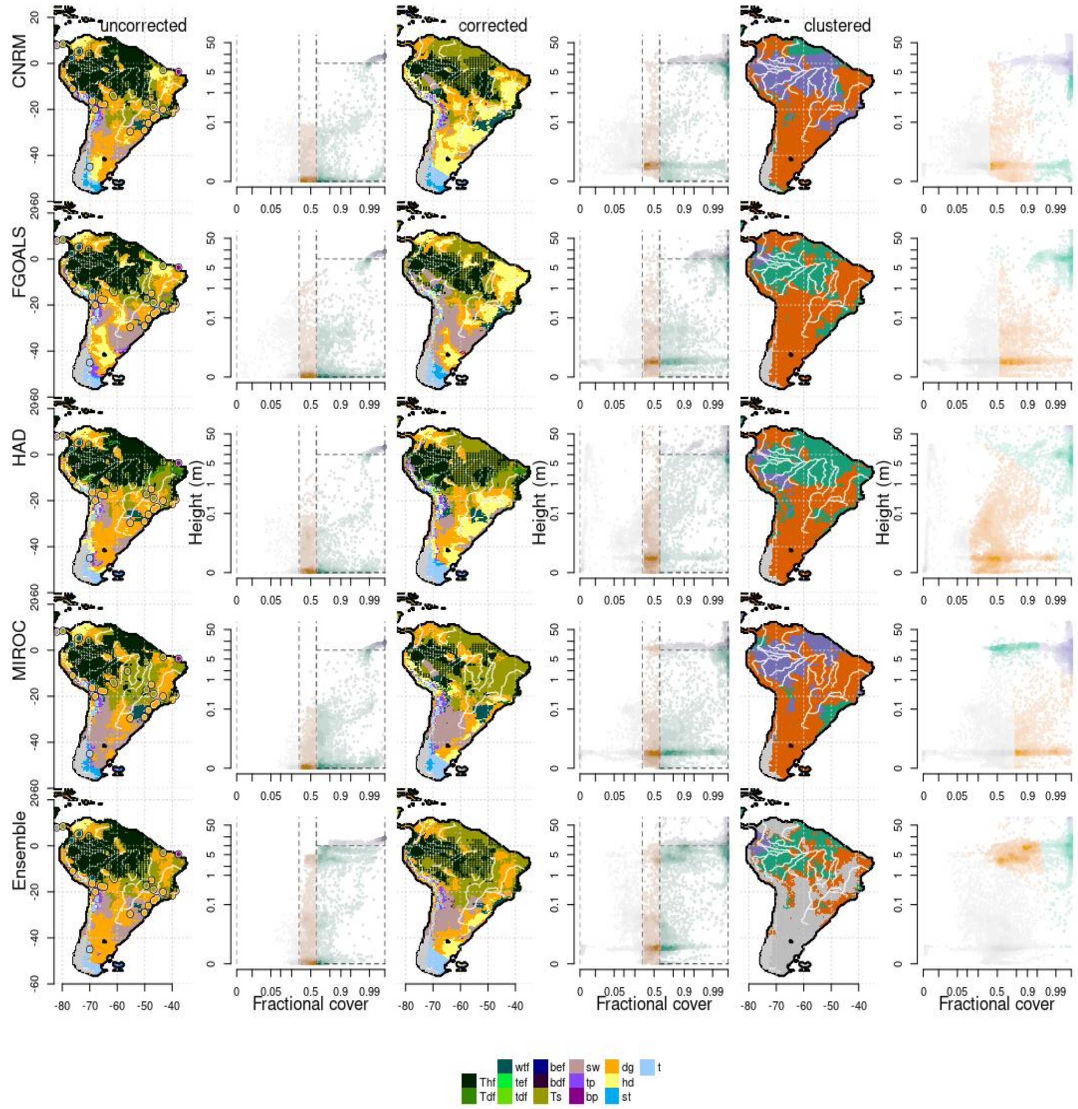

Supplementary Figure 9. All reconstructed biomes. As per Figure 1 and including (top to bottom) CNRM, FGOALS, HDGEM2, MIROC and the Ensemble model.

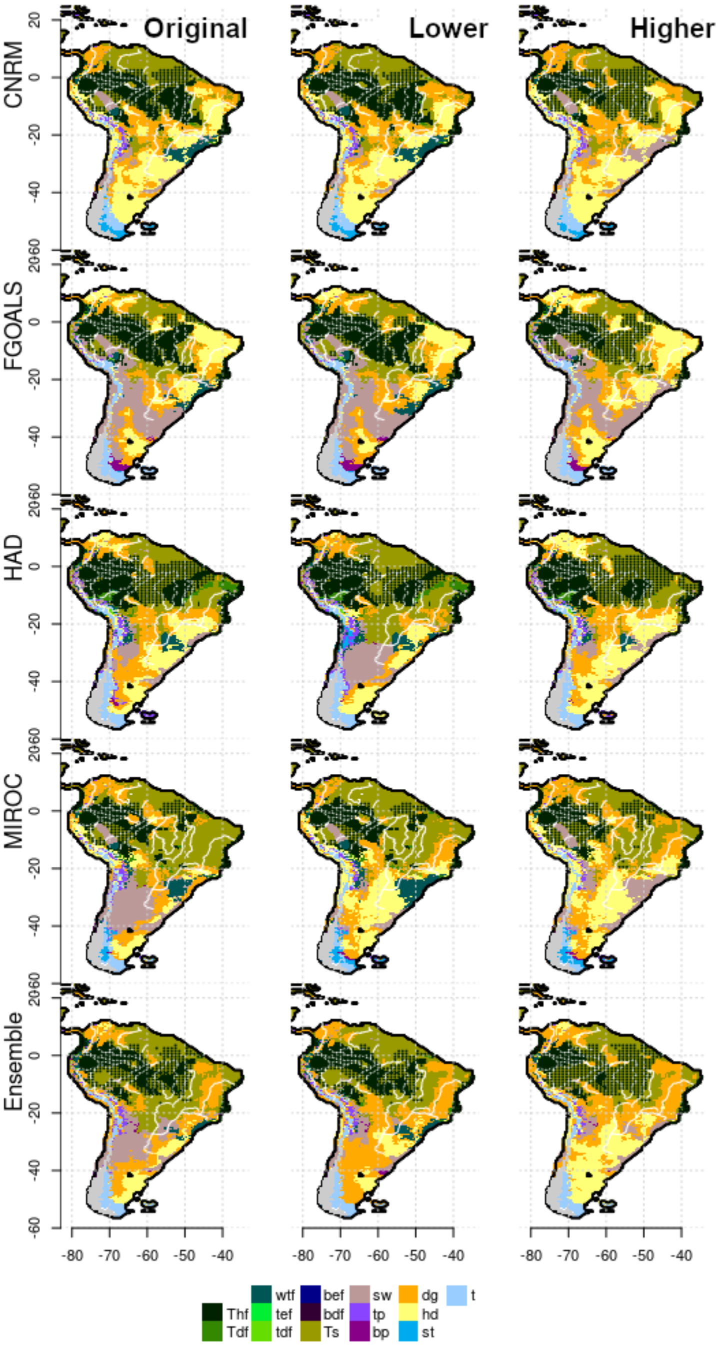

Supplementary Figure 10. Sensitivity of biome reconstructions to biomosation thresholds for Foliage Projected Cover (FPC) and height. First column shows bias-corrected biomes using the scheme as shown in Supplementary Figure 8. Second column bias-corrected biomes with FPC thresholds changes from 0.3, 0.6 to 0.2 and 0.4 and height from 5 and 10m to 2.5 and 5m. Third column bias-corrected biomes with FPC thresholds changes to 0.7 and 0.75 and height to 10 and 20m.
